# Hybrid YOLO-Inception-ResNetV2 Pipeline for Automated Human Activity Recognition in Controlled Environments

**DOI:** 10.7759/cureus.104927

**Published:** 2026-03-09

**Authors:** Pranay Mandadapu

**Affiliations:** 1 Computer Science, University of Wisconsin-Milwaukee, Milwaukee, USA

**Keywords:** computer vision, deep learning, human activity recognition, inception-resnetv2, patient safety monitoring

## Abstract

Human activity recognition (HAR) plays a vital role in healthcare monitoring, but manual video annotation remains a time-consuming and error-prone process. This study presents a hybrid deep learning pipeline that automates HAR from controlled environment videos by integrating You Only Look Once (YOLO)-based human detection with Inception-ResNetV2 classification. The system first employs YOLOv8 to isolate human subjects from the surrounding background and then uses separate models for cropped and uncropped frames, selected dynamically based on human detection. Experiments were conducted on a dataset of 18 subjects, each recorded for 12 hours in a metabolic chamber, representing real-world activities such as sitting, standing, walking, and lying. The hybrid approach achieved an overall accuracy of 66.11%, improving by 9.43% compared to the baseline model without YOLO preprocessing. These results show that isolating human subjects improves classification performance (66.11% accuracy, +9.43% over baseline), although overall accuracy remains modest and is influenced by class imbalance. The findings suggest potential for automated activity monitoring in controlled healthcare environments.

## Introduction

Human activity recognition (HAR) is a pivotal technology in computer vision and machine learning, enabling automated identification of human actions from video or sensor data [1]. Its applications span diverse domains, including surveillance, sports analytics, and, notably, healthcare, where continuous monitoring of patient activities can enhance safety and care efficiency [2]. In settings such as nursing homes and hospitals, HAR systems promise to alleviate the burden of manual observation, ensuring timely interventions while reducing staff workload. However, a significant bottleneck in video-based HAR is the reliance on manual annotation, a process that is labor-intensive, costly, and prone to errors. For instance, annotating 12-hour video recordings of multiple subjects, as in our dataset, requires meticulous labeling of diverse activities such as sitting, standing, walking, and lying, making scalability a critical challenge.

Traditional HAR approaches often leverage convolutional neural networks (CNNs) and pretrained models like ResNet50 to extract spatial features from video frames [3]. While effective, these methods struggle with visual noise from complex backgrounds, which can obscure relevant human actions and degrade classification accuracy. Recent advancements in object detection, such as the You Only Look Once (YOLO) framework [4,5], offer robust solutions for isolating objects of interest, yet their integration into HAR pipelines remains underexplored, particularly in controlled environments like metabolic chambers. This research evaluates a hybrid pipeline that combines YOLOv8-based human detection with Inception-ResNetV2 classification, specifically assessing its performance on a controlled metabolic-chamber dataset. This study evaluates the proposed pipeline as a feasibility demonstration using healthy subjects in a controlled metabolic chamber environment, serving as a first step toward potential future translation to clinical populations and real-world healthcare settings.

The proposed approach introduces a dual-model strategy: one model is trained on original video frames, while another is trained on cropped images where human subjects are isolated from their environment using YOLOv8. This dynamic selection, based on whether a human is detected in a frame, reduces background noise and focuses the classification process on relevant features. This frame-based approach performs posture-level recognition, which is appropriate for the slowly changing activities (sitting, standing, walking) observed in the controlled metabolic chamber environment. We evaluate this pipeline on a dataset comprising 12-hour video recordings of 18 subjects in a metabolic chamber, capturing activities of daily living. The methodology employs pre-trained deep residual networks (ResNet50, ResNet152V2, and Inception-ResNetV2) and incorporates leave-one-subject-out cross-validation and class weighting to address dataset imbalances. This article was previously posted to the TechRxiv preprint server on October 30, 2025.

Background

HAR systems are broadly categorized into vision-based and sensor-based approaches. Vision-based HAR, the focus of this work, relies on video or image data to classify activities, offering rich contextual information but posing computational challenges due to complex backgrounds and varying viewpoints [1]. Sensor-based HAR, using wearables or Internet of Things (IoT) devices, is less computationally intensive but limited by sensor placement and data granularity [2]. Visual noise from cluttered environments often degrades model performance, a challenge our work addresses through human isolation using YOLOv8.

Deep learning has revolutionized HAR by enabling end-to-end feature learning from raw data, reducing the need for manual preprocessing [6]. CNNs are widely used for spatial feature extraction in video-based HAR. For instance, He et al. introduced ResNet architectures, which leverage residual connections to train deep networks effectively, achieving state-of-the-art performance on image recognition tasks [3]. These models, including ResNet50 and ResNet152V2, are commonly fine-tuned for HAR tasks due to their representational power [7]. Inception-ResNetV2, combining Inception modules with residual connections, further enhances feature extraction at multiple scales [8]. However, these models often struggle with irrelevant background information in video frames, which can obscure human actions. Recurrent neural networks (RNNs) have also been explored for modeling temporal dependencies in HAR [9], though they are computationally expensive for long videos. Our approach mitigates this by preprocessing frames with YOLOv8 to isolate human subjects, improving classification accuracy. In line with our hypothesis that foreground isolation reduces background noise and improves classification robustness, we adopted single-frame sampling at one-minute intervals, which is appropriate for the slowly evolving activities in the controlled metabolic chamber environment.

Object detection in HAR

Object detection frameworks like YOLO have transformed computer vision by enabling real-time, accurate object localization [4,5]. YOLOv3 and YOLOv8, trained on large datasets like Open Images V7 [10], excel at detecting objects across scales, including humans in complex scenes. While YOLO has been used in surveillance and autonomous systems, its application in HAR, particularly for isolating humans to reduce background noise, is less explored. Our work pioneers this integration by employing YOLOv8 to crop human subjects from video frames, feeding them into a separate Inception-ResNetV2 model for activity classification.

Clinical relevance

HAR's application in healthcare, particularly within the Internet of Healthcare Things (IoHT), is a growing area of interest. Zhou et al. propose a semi-supervised deep learning framework for IoHT, leveraging multimodal sensor data to enhance HAR accuracy in healthcare settings [2]. Their work underscores the potential of automated monitoring in nursing homes and hospitals, where continuous observation is critical but resource-intensive. Similarly, Hartmann et al. advocate incorporating physiological knowledge into HAR models to improve robustness [11]. Despite these advancements, existing HAR methods often overlook the impact of environmental noise in controlled video settings, where background elements can interfere with activity recognition. Our approach combines YOLOv8 for human isolation with Inception-ResNetV2 for classification, using a dual-model strategy that dynamically selects between cropped and uncropped frame models based on human detection. This method addresses scalability challenges in healthcare monitoring, offering a pathway for real-time, automated systems in controlled environments like metabolic chambers.

## Materials and methods

Dataset

The dataset, sourced from the Department of Kinesiology at the University of Wisconsin-Milwaukee, comprises 12-hour video recordings of 18 healthy subjects performing activities of daily living in a metabolic chamber, a controlled environment designed to measure energy consumption [12]. Activities include sitting, standing, walking, lying, and others (e.g., crouching, kneeling), totaling 216 hours of footage. Frames were extracted at one-minute intervals to reduce computational load while preserving sufficient temporal information for activity recognition. The dataset was manually annotated, categorizing activities into four primary classes: sitting, standing, walking, and "other" (grouping less frequent activities like lying due to their minimal representation).

**Figure 1:**
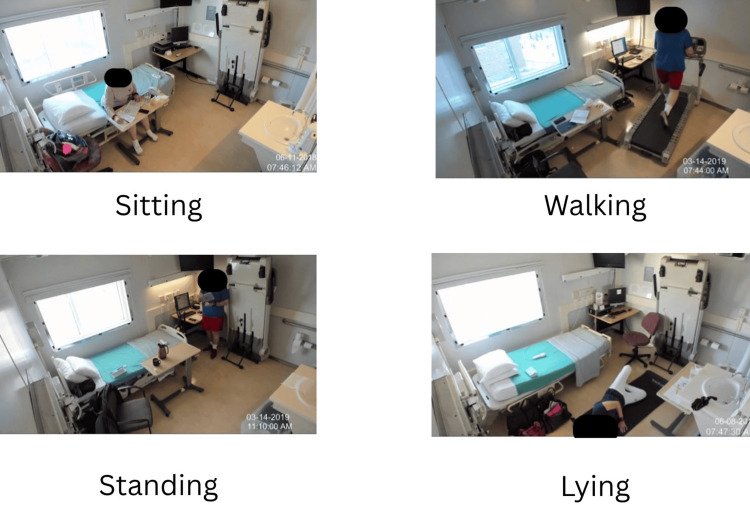
Collage of different subjects doing different activities The figure was created by the author from photographs taken from the dataset collected in the metabolic chamber at the University of Wisconsin-Milwaukee.

Figure 1 illustrates sample frames, showing subjects engaged in sitting, walking, standing, and lying activities. Table 1 details the class distribution across subjects, revealing a significant imbalance, with sitting dominating (e.g., 54.95% for subject CO1001) and "other" activities comprising only 6.97% on average. This severe class imbalance (~69% sitting overall) is a central limitation of the study that largely explains the reported accuracy and was only partially mitigated by class weighting during training.

**Table 1:** Data distribution of uncropped images among different classes and subjects Data are presented as percentages (%).

Subject ID	Sitting	Standing	Walking	Other
CO1001	54.95%	25.80%	12.27%	6.97%
CO1002	44.67%	30.98%	7.88%	16.46%
CO1007	81.13%	13.22%	0.41%	5.23%
CO1008	78.75%	11.67%	7.78%	1.81%
CO1009	72.26%	13.18%	13.18%	1.39%
CO1010	55.39%	23.62%	12.02%	8.98%
CO1011	76.67%	17.64%	4.44%	1.25%
CO1025	71.13%	22.04%	5.58%	1.26%
CO1031	60.64%	16.13%	4.31%	18.92%
CO1063	81.55%	6.10%	9.57%	2.77%
CO1067	78.53%	13.57%	3.32%	4.57%
CO1069	64.71%	17.43%	10.32%	7.53%
CO1070	66.02%	28.85%	2.91%	2.22%
CO1072	82.45%	10.31%	3.34%	3.90%
CO1073	31.43%	49.65%	12.80%	6.12%
CO1074	90.81%	4.04%	4.18%	0.97%
CO1075	87.17%	8.79%	0.42%	3.63%
CO1076	68.51%	8.84%	8.56%	14.09%
Overall distribution	69.26%	17.88%	6.80%	6.0%

Data preprocessing

To manage the computational complexity of 216 hours of video, frames were extracted at one-minute intervals, resulting in a manageable dataset for training and evaluation. This one-minute sampling interval is appropriate for the slowly changing activities (sitting, standing, walking, and lying) in the controlled metabolic chamber, effectively performing posture-level recognition rather than capturing full temporal dynamics. YOLOv8, trained on Open Images V7 [10], was employed to detect and crop human subjects from each frame, creating a secondary dataset focused solely on human figures. This preprocessing reduces background noise, hypothesizing that isolated human figures enhance classification accuracy. If YOLOv8 fails to detect a human, the original (uncropped) frame is used, enabling a dual-model approach. Although the one-minute sampling interval and variable YOLO detection rates across subjects may introduce some selection bias, leave-one-subject-out cross-validation was used to promote generalization and mitigate subject-specific effects.

Model architecture

The proposed pipeline uses a hybrid approach with two models: one trained on original frames and another on YOLO-cropped frames. Three pre-trained deep residual networks, ResNet50, ResNet152V2, and Inception-ResNetV2, were evaluated, with weights initialized from ImageNet [13]. Each model was adapted for HAR by freezing base layers to retain learned features and adding custom layers: a Global Average Pooling layer, a Dense layer with L2 regularization and ReLU activation, a Dropout layer (to prevent overfitting), and a Dense output layer with softmax activation for classifying four activity classes. Inception-ResNetV2 was selected for final evaluations due to its balance of depth and performance [8]. The 9.43% accuracy improvement is attributed to the YOLOv8 cropping step rather than the choice of backbone architecture, as the same Inception-ResNetV2 model and custom head were used for both the cropped and uncropped pipelines.

**Figure 2:**
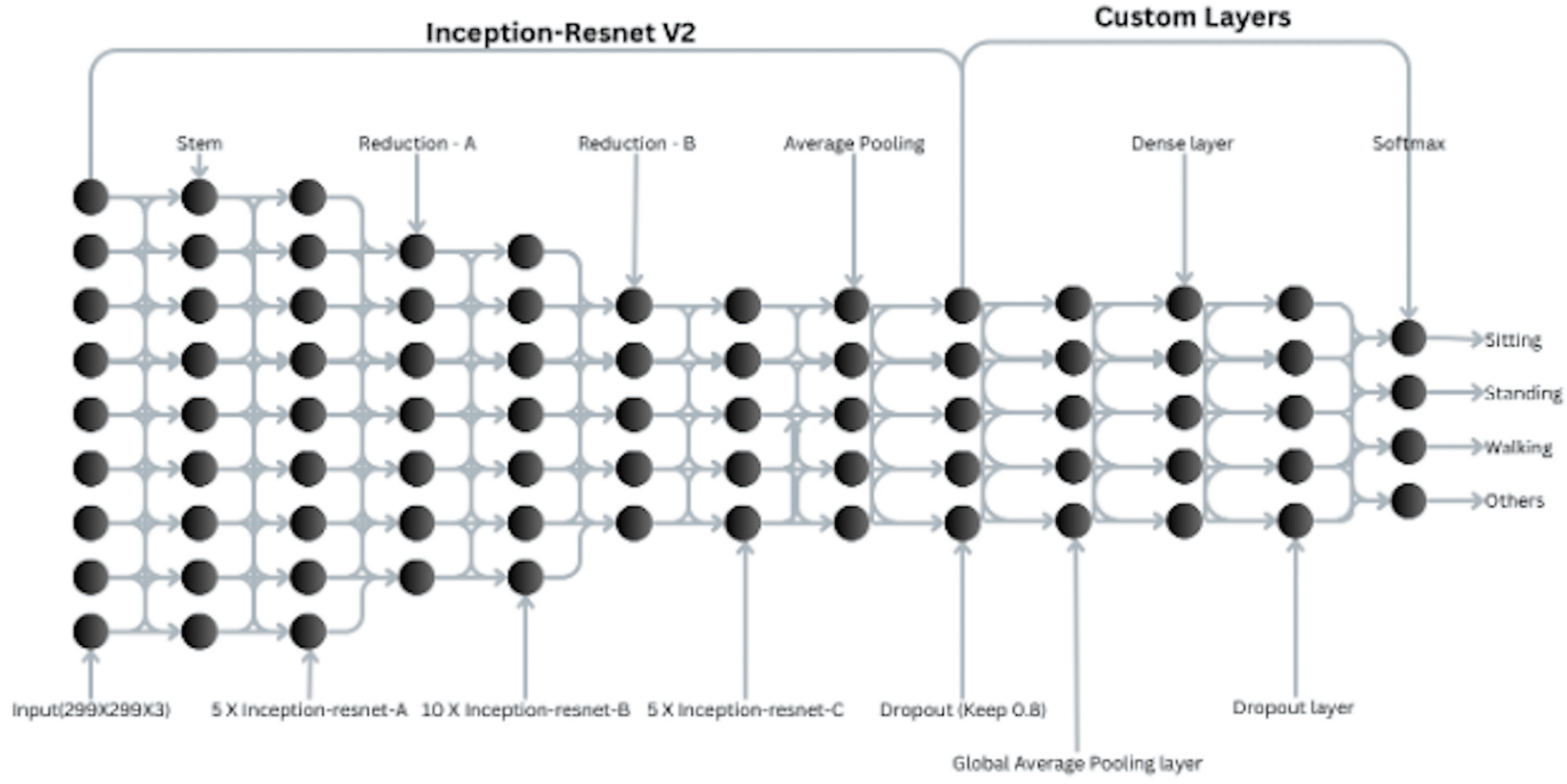
Architecture overview This is an original graphical illustration created by the author using Canva (Canva Pty Ltd., Sydney, Australia).

Figure 2 illustrates the architecture, highlighting the integration of YOLOv8 preprocessing with the classification model. The dual-model strategy dynamically selects the cropped-frame model when a human is detected, otherwise using the original-frame model.

Training models were trained using leave-one-subject-out cross-validation, where data from 17 subjects form the training set and one subject is reserved for testing, iterating across all subjects to ensure robust generalization. To address class imbalance, the compute_sample_weight function from scikit-learn was used to assign higher weights to minority classes (e.g., "other"), balancing their contribution to the loss function [14]. Training ran for 20 epochs with callbacks including ReduceLROnPlateau for adaptive learning rate adjustment, Model Checkpoint to save the best model, and Early Stopping to prevent overfitting. The Adam optimizer was used with a batch size of 32. Variance across folds and statistical confidence on the 9.43% improvement were not computed, as each leave-one-subject-out iteration was run once with fixed random seeds.

Evaluation metrics

Model performance was assessed using standard classification metrics: accuracy, precision, recall, and F1-score [15]. Due to severe class imbalance, per-class F1-scores were emphasized alongside overall accuracy when interpreting results. These metrics were computed per class and overall, with confusion matrices generated to analyze misclassifications, particularly for imbalanced classes like "other".

## Results

Evaluation of Inception-ResNetV2 without YOLO preprocessing

The Inception-ResNetV2 model, augmented with custom layers (Global Average Pooling, Dense with L2 regularization, Dropout, and softmax output), was evaluated on the original (uncropped) dataset using leave-one-subject-out cross-validation.

Table 2 presents the confusion matrix for four activity classes: others, sitting, standing, and walking. The model achieved an overall accuracy of 56.68%. Key metrics include others: recall of 16.30% and F1-score of 14.03%, indicating difficulty in recognizing less frequent activities due to class imbalance; sitting: high recall (69.05%) and F1-score (72.72%), reflecting strong performance on the dominant class; standing: moderate recall (32.88%) and F1-score (31.91%), suggesting challenges with partial visibility or background noise; and walking: recall of 49.33% and F1-score of 34.72%. Precision was highest for sitting (76.80%) and lowest for others (12.32%), highlighting the impact of class imbalance on minority classes. The results confirm a strong bias toward the dominant “sitting” class, with very low recall (16.30%) for the minority “others” category, indicating limited general recognition capability.

**Table 2:** Confusion matrix of Inception-ResNet V2 without YOLO image preprocessing Data are presented as numbers (n) for the confusion matrix counts and as percentages (%) for the evaluation metrics (Recall, Precision, F1-Score).

Confusion Matrix	Others	Sitting	Standing	Walking	Correct	Total	Recall
Others	127	383	182	87	127	779	16.30%
Sitting	685	6020	1441	833	6020	8979	69.05%
Standing	135	1143	762	278	762	2318	32.88%
Walking	84	292	74	438	438	888	49.33%
Correct	127	6020	762	438	7347		
Total	1031	7838	2459	1636		12,964	
Precision	12.32%	76.80%	30.99%	26.78%			Accuracy = 56.68%
F1-Score	14.03%	72.72%	31.91%	34.72%			

Evaluation of Inception-ResNetV2 with YOLO preprocessing

The hybrid pipeline, integrating YOLOv8 for human detection and cropping, was evaluated using a dual-model approach. Frames with detected humans were processed by a model trained on cropped images, while undetected frames used the original-frame model. While the hybrid pipeline achieved a 9.43% improvement in overall accuracy, the gains were concentrated in the dominant classes (sitting and standing), with recall for the minority “others” class remaining poor at 13.65%.

Table 3 shows the YOLO detection rates across subjects.

**Table 3:** YOLO detection rate from the original dataset Data are presented as percentages (%).

Subject ID	YOLO Detected Humans	Not Detected By YOLO
CO1001	83.57%	16.43%
CO1002	88.26%	11.74%
CO1007	75.1%	24.9%
CO1008	97.36%	2.64%
CO1009	65.93%	34.07%
CO1010	90.07%	9.93%
CO1011	77.67%	22.33%
CO1025	98.19%	1.81%
CO1031	67.78%	32.22%
CO1063	76.59%	23.41%
CO1067	74.55%	25.45%
CO1069	69.64%	30.36%
CO1070	91.69%	8.31%
CO1072	52.99%	47.01%
CO1073	90.69%	9.31%
CO1074	82.2%	17.8%
CO1075	60.45%	39.55%
CO1076	92.55%	7.45%

Table 4 presents the confusion matrix for the hybrid model. The hybrid model achieved an overall accuracy of 66.11%, a 9.43% improvement over the baseline. Key metrics include sitting: improved recall (75.89%) and F1-score (80.67%), benefiting from reduced background noise; standing: enhanced recall (54.87%) and F1-score (51.02%), showing better detection of human-focused features; walking: improved recall (58.79%) and F1-score (45.94%), indicating robustness in dynamic activities. Precision improved across all classes, particularly for sitting and standing, due to YOLO's noise reduction.

**Table 4:** Confusion matrix of Inception-ResNet V2 with YOLO image preprocessing Data are presented as numbers (n) for the confusion matrix counts and as percentages (%) for the evaluation metrics (Recall, Precision, F1-Score).

Confusion Matrix	Others	Sitting	Standing	Walking	Correct	Total	Recall
Others	138	509	189	175	138	1011	13.65%
Sitting	784	6638	934	391	6638	8747	75.89%
Standing	209	540	1272	297	1272	2318	54.87%
Walking	67	26	273	522	522	888	58.79%
Correct	138	6638	1272	522	8570		
Total	1198	7713	2668	1385		12964	
Precision	11.52%	86.07%	47.67%	37.69%			Accuracy = 66.11%
F1-Score	12.50%	80.67%	51.02%	45.94%			

Subject-wise evaluation

Subject-wise analysis revealed performance variability. High accuracies were observed for subjects like CO1074 (94.71%) and CO1063 (81.14%), where subjects wore brighter clothing and were centrally positioned. Lower accuracies for CO1031 (30.46%) and CO1073 (32.96%) were due to challenges like dark clothing blending with the background. This performance dependence on clothing brightness and visibility suggests the model relies heavily on low-level visual contrast rather than deeper semantic understanding of activities, highlighting a limitation in robustness.

**Figure 3:**
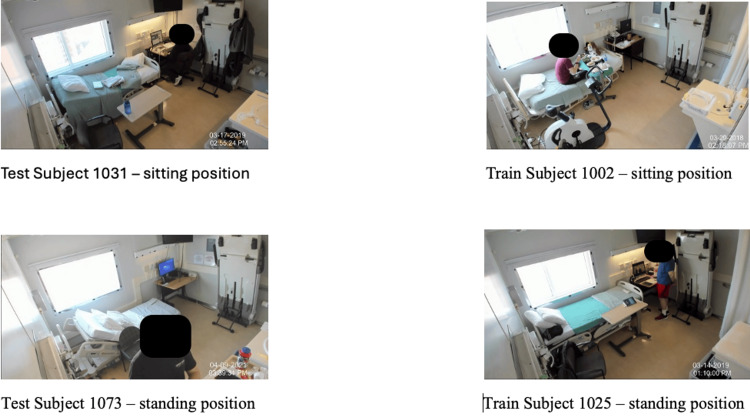
Test and train subject comparison The figure was created by the author from photographs taken from the dataset collected in the metabolic chamber at the University of Wisconsin-Milwaukee.

Figure 3 illustrates these challenges, comparing a subject with dark clothing blending into the background against a subject with clear visibility. Subject CO1074 achieved the highest accuracy (89.83%) with the hybrid model, while CO1031 remained the lowest (43.12%), partly due to YOLO detecting humans in only 67% of frames. This highlights the importance of robust detection for effective preprocessing.

Table 5 summarizes the subject-wise accuracy improvement with the hybrid model. The overall 9.43% accuracy improvement demonstrates the efficacy of YOLOv8 preprocessing in reducing background noise, particularly for sitting and standing activities.

**Table 5:** Subject-wise accuracy with YOLO image preprocessing Data are presented as percentages (%).

Hybrid	Accuracy
CO1001	68.4797768%
CO1002	63.4854772%
CO1007	59.2286501%
CO1008	85%
CO1009	70.1803051%
CO1010	65.6077348%
CO1011	75.1388889%
CO1025	58.4379358%
CO1031	43.1154381%
CO1063	61.1650485%
CO1067	77.9778393%
CO1069	68.0613668%
CO1070	71.0124827%
CO1072	62.3955432%
CO1073	57.7190542%
CO1074	89.8328691%
CO1075	67.5034868%
CO1076	68.5082873%

## Discussion

The hybrid YOLO-Inception-ResNetV2 pipeline significantly enhances HAR performance, achieving a 66.11% overall accuracy compared to the 56.68% baseline without YOLO preprocessing, a 9.43% improvement [1]. This gain is primarily due to YOLOv8's ability to isolate human subjects, reducing background noise in video frames from controlled environments like metabolic chambers. The model excelled in recognizing dominant activities, with recall improvements for sitting (75.89% vs. 69.05%) and standing (54.87% vs. 32.88%), as the cropped dataset focuses on human-specific features [1]. These results underscore the efficacy of the dual-model strategy, where frames with detected humans leverage a model trained on cropped images, while undetected frames use the original-frame model, enhancing robustness across varied scenarios.

However, challenges remain, particularly for the "others" category, which achieved a low recall (13.65%) and F1-score (12.50%) due to class imbalance [1]. Subjects like CO1031 exhibited lower accuracy (43.12% with YOLO) because YOLOv8 detected humans in only 67% of frames, compounded by dark clothing blending with backgrounds [1]. This highlights the dependency on robust human detection for the hybrid approach's success. The controlled environment of the metabolic chamber, while ideal for testing, limits generalizability to real-world settings with diverse lighting and backgrounds. These findings suggest that while the pipeline is a promising step toward scalable HAR systems, further refinements are needed to handle imbalanced datasets and varied environments. The frame-based approach also represents a major limitation, as it omits temporal modeling of activity dynamics.

Clinical implications

The implications for healthcare are substantial. Automated HAR systems can reduce the workload of manual monitoring in nursing homes and hospitals, enabling real-time patient observation and timely interventions [2]. The proposed pipeline's ability to mitigate environmental noise makes it suitable for controlled settings, paving the way for integration into the IoHT [2]. By leveraging transfer learning with pre-trained models like Inception-ResNetV2, the approach balances computational efficiency with high accuracy, making it feasible for resource-constrained healthcare facilities. These statements represent potential applicability rather than demonstrated clinical performance, as the current study was conducted solely in a controlled non-clinical metabolic chamber without real-time inference, workflow simulation, or diverse real-world environments.

Limitations

Despite these promising results, this study has limitations. The dataset exhibits class imbalance, which impacted performance on minority categories. Additionally, the data was collected in a controlled metabolic chamber, which may not fully represent the variability of real-world environments with diverse lighting and cluttered backgrounds. Future work should explore advanced techniques for handling imbalanced datasets, such as oversampling or generative methods, and expand the dataset to include diverse environments to improve generalizability. Additional key limitations include the frame-level (non-temporal) modeling, the small sample size of 18 subjects, the heuristic dual-model switching strategy, and the lack of an external validation dataset.

## Conclusions

This study proposed a hybrid pipeline integrating YOLO-based human detection with deep residual networks to automate HAR. By isolating human subjects from their environment, the system demonstrated that reducing background noise significantly enhances classification reliability, particularly for dominant activities like sitting and standing. These findings underscore the potential of combining object detection with activity classification to create more robust monitoring systems. Ultimately, this approach offers a scalable pathway for improving patient safety and operational efficiency in healthcare settings, such as nursing homes and assisted living facilities, where automated, non-invasive monitoring is increasingly critical. Overall, the work serves as a feasibility demonstration that human-centric cropping can modestly improve classification performance under the constrained conditions of a controlled metabolic chamber.
